# Equal-Spin Andreev Reflection on Junctions of Spin-Resolved Quantum Hall Bulk State and Spin-Singlet Superconductor

**DOI:** 10.1038/s41598-018-21707-0

**Published:** 2018-02-22

**Authors:** Sadashige Matsuo, Kento Ueda, Shoji Baba, Hiroshi Kamata, Mizuki Tateno, Javad Shabani, Christopher J. Palmstrøm, Seigo Tarucha

**Affiliations:** 1 Department of Applied Physics, University of Tokyo, 7-3-1 Hongo, Bunkyo-ku, Tokyo, 113-8656 Japan; 2 Center for Emergent Matter Science, RIKEN, 2-1, Hirosawa, Wako-shi, Saitama, 351-0198 Japan; 3 California NanoSystems Institute, University of California, Santa Barbara, California 93106 USA; 4 Center for Quantum Phenomena, Physics Department, New York University, NY, 10003 USA; 5 Electrical and Computer Engineering, University of California, Santa Barbara, California 93106 USA; 6 Materials Department, University of California, Santa Barbara, California 93106 USA

## Abstract

The recent development of superconducting spintronics has revealed the spin-triplet superconducting proximity effect from a spin-singlet superconductor into a spin-polarized normal metal. In addition recently superconducting junctions using semiconductors are in demand for highly controlled experiments to engineer topological superconductivity. Here we report experimental observation of Andreev reflection in junctions of spin-resolved quantum Hall (QH) states in an InAs quantum well and the spin-singlet superconductor NbTi. The measured conductance indicates a sub-gap feature and two peaks on the outer side of the sub-gap feature in the QH plateau-transition regime increases. The observed structures can be explained by considering transport with Andreev reflection from two channels, one originating from equal-spin Andreev reflection intermediated by spin-flip processes and second arising from normal Andreev reflection. This result indicates the possibility to induce the superconducting proximity gap in the the QH bulk state, and the possibility for the development of superconducting spintronics in semiconductor devices.

## Introduction

A junction of superconductor and normal metal is a platform to observe superconducting proximity effect, in which the superconducting property penetrates to the normal metal. In a microscopic description of the proximity effect, an electron in the normal metal enters the spin-singlet superconductor, forming a Cooper pair with another electron with opposite spin, reflecting a hole into the normal metal, in a process called Andreev reflection (AR)^1^. In this picture, no AR is expected in the case of a fully spin polarized normal metal, however recently, theoretical and experimental studies in junctions with spin-polarized normal metal, revealed existence of the spin-triplet superconducting proximity effect^2–8^. The spin-triplet proximity effect is only allowed when spin-flip processes intermediate “equal-spin” AR, which is possible due to the presence of magnetization inhomogeneity or spin-orbit interaction^9,10^. Ferromagnetic metal has been utilized in experiments of superconducting spintronics to date. A semiconductor material however offers several advantages, including the control of carrier density and spin filling through electrical gating and magnetic field, and the possibility of ballistic transport in micron sized devices. Furthermore strong spin-orbit interaction can be utilized in two dimensional electron gases (2DEGs) in narrow gap semiconductors such as InAs and InSb^11–14^. These features favor the formation of spin-polarized states when the 2DEG is in the quantum Hall (QH) regime. Indeed, there are several experimental reports focusing on superconductor-semiconductor junctions in the QH regime^15–20^. However, all of these experiments have focused on the spin-degenerate QH states and the spin-triplet proximity effect has yet to be experimentally addressed, despite theoretical predictions of spin-triplet supercurrent in Josephson junctions with weak links of spin resolved QH edge channels^10^. Additionally, if the superconducting proximity gap is induced into the spin-resolved QH state, the system can be a topological superconductor^21^ and give a new platform to realize the Majorana Fermions^22,23^ whose signatures have recently been reported^24–31^.

Here we report an experimental study on electron transport in junctions between spin-resolved QH states and spin-singlet superconductors. We prepared junctions consisting of a high mobility InAs quantum well (QW) with NbTi contacts. The NbTi layers are contacted to the sides of the mesa containing the QW, minimizing the damage to the 2DEG. The 2DEG possesses a large g-factor, high mobility, and strong spin-orbit interaction, all necessary ingredients for coexistence of superconductivity and spin-resolved QH states. We observe spin-resolved quantized steps at magnetic fields below the superconducting critical field, 7 T, and find that the differential conductance has a dip or a peak structure as a sub-gap feature in all QH plateau-transition regimes of filling factor between 0 to 4. Additionally, we find two side peaks on the outer side of the sub-gap feature. We conclude that the structures observed here are a result of the equal-spin AR between the spin-resolved QH bulk states and the superconductor.

**Figure 1:**
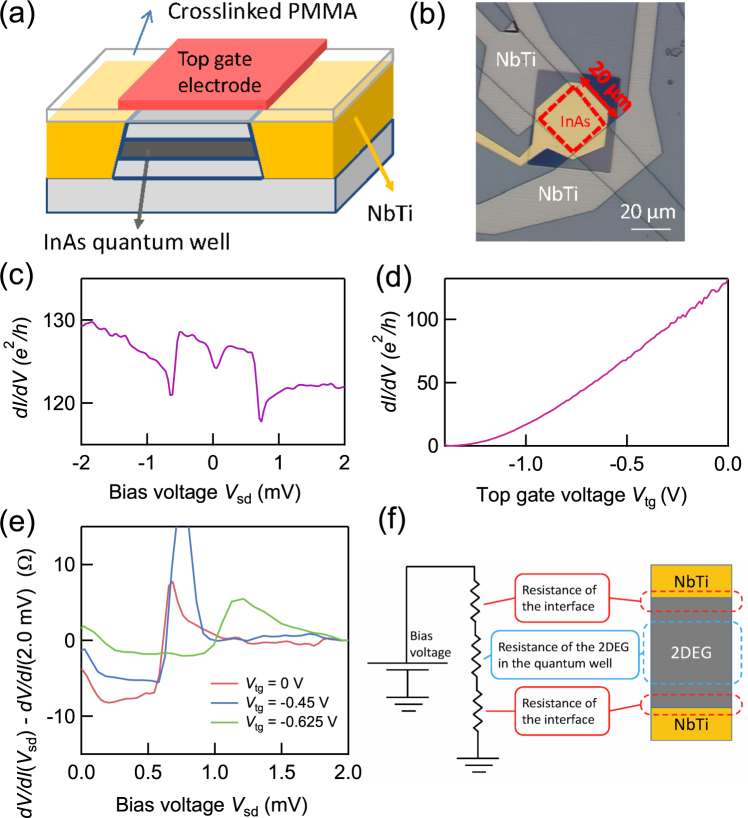
Device structure and the electron transport at *B* = 0 T. (**a**) Cross section of the fabricated device. The edges of the InAs QW are contacted with sputtered NbTi. (**b**) Optical image of the device. The region surrounded by the red dash line represents the mesa. (**c**) *dI*/*dV* vs. *V*_sd_ at *V*_tg_ = 0 V and *B* = 0 T. *dI*/*dV* measured in the range −0.71 m*V* < *V*_sd_ < 0.71 mV is enhanced due to AR. (**d**) *dI*/*dV* vs. *V*_tg_ at *V*_sd_ = 0 V is shown. The InAs QW is completely depleted by *V*_tg_. (**e**) *dV*/*dI* with *dV*/*dI* measured at *V*_sd_ = 2.0 mV subtracted as a function of *V*_sd_ is shown. The red, blue, and green lines are measured at *V*_tg_ = 0, −0.45, and −0.625 V, respectively. The resistance reduction due to AR decreases as *V*_tg_ decreases, indicating that *V*_tg_ tunes the carrier density of not only the center region of the 2DEG but also region near the junctions. (**f**) Schematic image of an equivalent circuit to our junction devices. The applied *V*_sd_ is divided between the two junctions and the 2DEG.

We fabricated junction devices from an InAs QW grown by molecular beam epitaxy^32,33^ with carrier density 3 × 10^11^ cm^−2^ and mobility 3 × 10^5^ cm^2^*V*^−1^*s*^−1^. (The material stack is represented in Supplementary Information (SI)). The cross section of the device is schematically represented in Fig. 1(a). Sputtered NbTi with the critical field of 7.0 T and the critical temperature of 6.5 K contacts the edge of the 2DEG and a top gate structure is fabricated using an insulating layer of cross-linked PMMA (see SI for details). The optical microscope photo in Fig. 1(b) shows the top view of the device. The two junctions are separated by 20 *μ*m, so this device is assumed to have two independent junctions. We measured the two-terminal differential conductance in a He3He4 dilution refridgerator with a base temperature of 50 mK.

Figure 1(c) shows the measured *dI*/*dV* vs. *V*_sd_ at *B* = 0 T. The *dI*/*dV* is enhanced in the range of −0.71 mV < *V*_sd_ < 0.71 mV. This conductance enhancement arises from AR^1,34^ and only appears for the bias voltage in the junction less than the superconducting bulk gap energy Δ_*bulk*_. Therefore, we evaluate the Δ_*bulk*_ as approximately 0.35 meV. The observation of the sub-gap feature guarantees that the junction has enough quality to study AR between the superconductor and spin-resolved QH state.

Figure 1(d) shows *dI*/*dV* vs. the top gate voltage, *V*_tg_. *dI*/*dV* gradually decreases with decreasing *V*_tg_ and becomes pinched off for *V*_tg_ < −1.38 V. This indicates that the top gating efficiently varies the 2DEG carrier density but not necessarily near the junction. We measured *dI*/*dV* vs. *V*_sd_ for various values of *V*_tg_ to examine the effect of top gating on the sub-gap conductance. We derived the differential resistance as given by *dV*/*dI* = (*dI*/*dV*)^−1^ and then subtracted *dV*/*dI* measured at *V*_sd_ = 2.0 mV to eliminate the normal state resistance including a series resistance due to the InAs QW away from the junction. The result is shown in Fig. 1(e) for three different values of *V*_tg_ = 0, −0.45, and −0.625 V by the purple, blue, and green curve, respectively. It is clear to see that *dV*/*dI*(*V*_sd_) − *dV*/*dI*(2.0 m*V*) displays a pronounced reduction (or conductance enhancement) within the gap, and the reduction increases as *V*_tg_ is made more positive. If the top gating only varies the carrier density away from the junction, the *dV*/*dI* reduction below the gap should be constant with *V*_tg_. Therefore, the result of Fig. 1(e) indicates that the top gating is efficient enough to vary the carrier density in the InAs QW near the superconducting junction. In Fig. 1(e), *V*_sd_ to characterize the superconducting gap decreases as *V*_tg_ is made more positive. The *V*_sd_ shift is due to the change in the voltage dropped over the junctions when the series resistance of the 2DEG is altered. Schematics of the equivalent circuit for the device are shown in Fig. 1(f). In the constant voltage bias measurement, the effective junction voltage decreases as the carrier density of the QW decreases with decreasing *V*_tg_. Herein the applied voltage of *V*_sd_ is assumed to only drop across the junctions in the saturation region. Therefore we evaluate ${{\rm{\Delta }}}_{bulk}\simeq 0.35\,{\rm{meV}}$.

**Figure 2:**
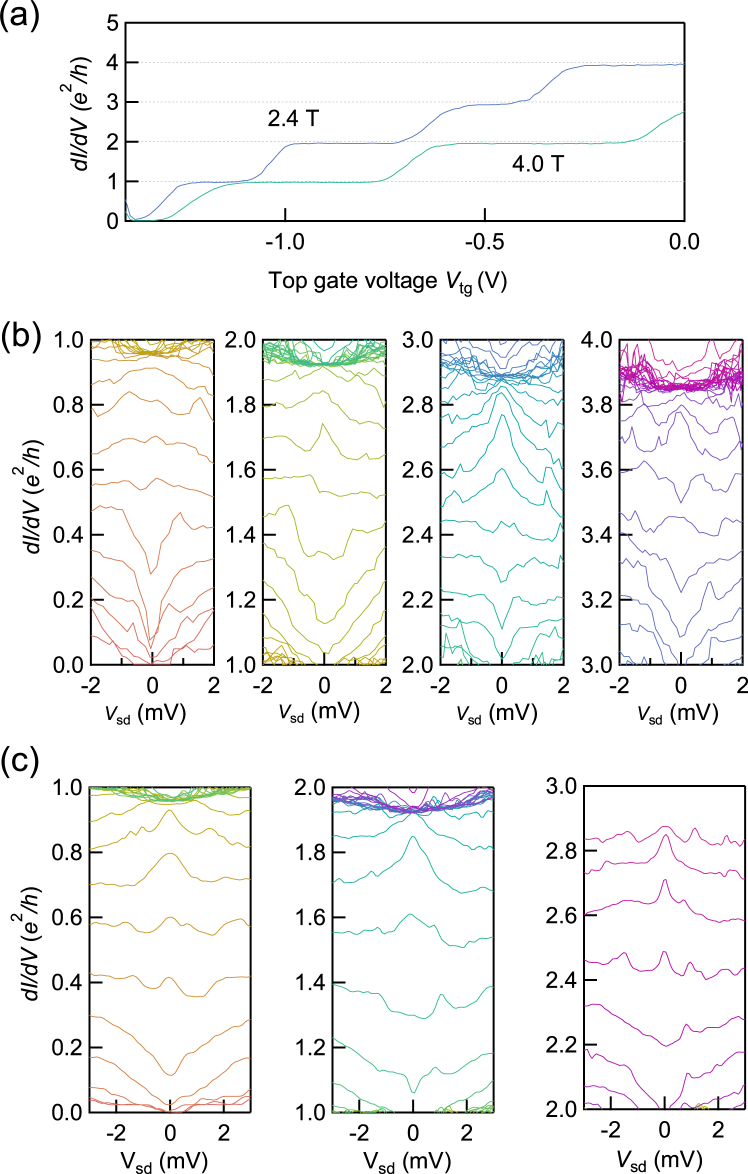
Measured spin-resolved QH effect ane sub-gap features on the QH plateau transition regimes at *B* = 2.4 T and 4 T. (**a**) *dI*/*dV* vs. *V*_tg_ at 2.4 T and 4.0 T with *V*_sd_ = 0 V. The conductance plateaus are clearly observed on 1, 2, 3, and 4 × *e*^2^/*h*. This indicates the Zeeman energy at 2.4 T is enough to resolve the spin degeneracy. (**b**) Measured *dI*/*dV* vs. *V*_sd_ at 2.4 T for −1.5 V < *V*_tg_ < 0 V, divided into four panels to clarify the *V*_sd_ dependence in each plateau-transition regime. *dI*/*dV* has a dip structure around *V*_sd_ = 0 V for *ne*^2^/*h* < *dI*/*dV* < *ne*^2^/*h* + 0.5. In contrast *dI*/*dV* has a peak structure for *ne*^2^/*h* + 0.5 < *dI*/*dV* < *ne*^2^/*h* + 0.8. (**c**) Measured *dI*/*dV* vs. *V*_sd_ at 4.0 T for −1.5 V < *V*_tg_ < 0 V divided into three panels. The features are similar to those observed in the results obtained at 2.4 T.

In Fig. 2(a), we present plots of measured *dI*/*dV* as a function of *V*_tg_ at out-of-plane magnetic field *B* = 2.4 and 4.0 T. The well-defined plateaus at integer multiples of *e*^2^/*h* which originate from the QH edge transport are clearly seen. From this observation, it is confirmed that the applied fields are strong enough to resolve the spin degeneracy, but significantly smaller than the critical field of the NbTi, implying that the superconductivity and the spin-resolved QH states coexist. We note that the spin-orbit energy *α* ⋅ *σ* × *k* can be estimated as 0.3 meV with *α* = 10^−10^ eVm^14,35^. This energy is much smaller than Zeeman energy at 4 T, 10 meV (see SI). Consequently, we ignore the effective field from the spin-orbit interaction in the spin-resolved QH state.

**Figure 3:**
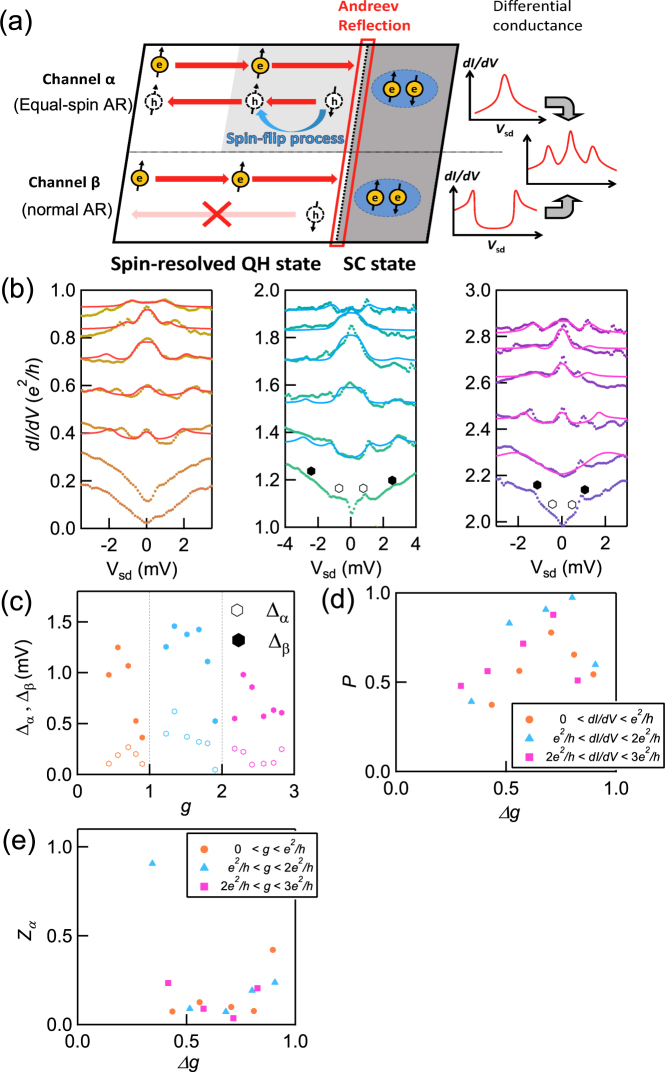
Numerical calculation results and the estimated parameters. (**a**) Schematic of AR in the channel *α* and *β*. AR in channel *α* is intermediated by the spin-flip process, while the reflection in the channel *β* is not. (**b**) Measured *dI*/*dV* vs. *V*_sd_ at 4.0 T indicated with dots with fitting results shown as solid lines. The open and closed hexagons are the position of Δ_*α*_ and Δ_*β*_ without the numerical fitting. (**c**) Obtained Δ_*α*_ and Δ_*β*_ are shown as open and closed hexagons, respectively. Δ_*β*_ has a convex upward trend in the respective plateau-transition regime. (**d**) *P*, indicating the relative contribution of the two channels, is shown as a function of Δ*g*. The orange circles, blue triangles and purple squares are *P* calculated from the analysis of the left, center, and right panels. (**e**) *Z*_*α*_ is shown as a function of Δ*g*. The position where *Z*_*α*_ has the minimum is the same as the *P*.

*dI*/*dV* vs. *V*_sd_ measured in the range of *V*_tg_ between −1.5 V and 0 V is represented in Fig. 2(b), and (c) for *B* = 2.4 T, and 4.0 T, respectively. *dI*/*dV* traces measured for magnetic fields spanning the conductance range *ne*^2^/*h* to (*n* + 1)*e*^2^/*h* with *n* = 0, 1, 2... are shown in the separate panels to highlight the sub-gap structure in the transition regions between plateaus. For example, *dI*/*dV* at *B* = 2.4 T between 0 < *dI*/*dV* < *e*^2^/*h* is shown in the leftmost panel of Fig. 3(b).

In the transition regions between the conductance plateaus, we find pronounced sub-gap features appearing as a dip, a peak and then a dip at around *V*_sd_ = 0 V from the lower to the upper plateau in all panels. Similar sub-gap features are previously reported for the junctions of two NbN superconductors and a spin-degenerated QH state in an InAs QW^15^. However, the underlying physics of the sub-gap feature remains to be elucidated. More interestingly in Fig. 2(b) and (c), the center peak appears broad in some traces and even split into two in others. In addition to a center dip or peak structure we observe a side peak. For example, it is clear to see two side peaks at *V*_sd_ = ±1.8 mV in addition to a peak at *V*_sd_ = 0 V for the curves at $dI/dV\simeq 2.5{e}^{2}/h$ in the right panel of Fig. 2(c).

The sub-gap conductance enhancement indicated by the observation of a zero-bias peak can be assigned to AR in the junction having a low potential barrier. In contrast, if the potential barrier is so large that normal reflection is more dominant than AR, a dip rather than a peak can appear according to the Blonder, Tinkham, and Klapwijk (BTK) theory^34^. Therefore we assign the peak (dip) structure observed in the plateau-transition regime to AR (normal reflection), and then the change of the sub-gap feature in Fig. 2(b) and (c) can be simply explained by considering the change of the junction potential barrier depending on *V*_tg_. For the transport through the QH state it is well established that the dominant contribution arises from the QH bulk state in the plateau-transition regime and from the QH edge state in the plateau regime. Herein, we deduce that the sub-gap feature, especially the peak structure is originated from AR in the junction between the superconductor and the spin-resolved QH bulk state. A finite amount of equal-spin AR can be expected for a 2DEG with strong spin-orbit interaction according to recent theoretical studies^9,10^. Therefore, we here assume coexistence of equal-spin AR and normal AR, corresponding to AR intermediated with and without a spin-flip process respectively, as schematically shown in Fig. 3(a).

This kind of sub-gap features might emerge due to the other origins. We discuss some of the origins here. First, recent theory predicts that the weak (anti)localization effect in the junction of a disordered normal metal and a superconductor^36^. This effect is originated from closed trajectory of an electron and a retro-reflected hole without consideration of any orbital motion invoked by the out-of-plane magnetic field. However, the orbital motion prevents the electron and the hole from making the closed trajectory. Therefore, this scenario is not assigned to the observed sub-gap feature. Second, the conductance enhancement can also be obtaind in the Kondo effect on the quantum dot. This is also not assigned the measured enhancement because our device structure is not a 0 or 1-dimensional but 2-dimensional system where the Kondo effect should appear as the resistance enhancement. Additionally the Kondo effect can easily collapse due to large Zeeman effect. Therefore we do not assume that the zero-bias peak is due to the Kondo effect. Third, the similar sub-gap features observed in junctions of superconductor NbN and an InAs nanowire were reported^37^. In this paper, the transient resonance is discussed as one of possible origins of the observed sub-gap features. The resonances arise when reflections at the interface between the bare segment and the NbTi-covered segments induce constructive interference. Our data shows the clear trend of the sub-gap feature; the peak structure appears only in the plateau transition regime. Additionally, this trend reproduces at 2.4 T and 4.0 T, which give different cyclotron radius (<100 nm). This clear trend observed in the several QH plateau transition regimes at the different magnetic fields means that the sub-gap feature is strongly related to the QH effect, not the detail of the interface.

In order to more quantitatively interpret the sub-gap features including the side peaks, we construct a model based on the BTK theory. In this theory, the normalized differential conductance of the junction, *G*_int_(*V*_sd_,*Z*,*T*,Δ), can be written as

1 $${G}_{{\rm{int}}}({V}_{{\rm{sd}}},Z,T,{\rm{\Delta }})={\int }_{-\infty }^{\infty }\frac{df(E-{V}_{{\rm{sd}}},T)}{d{V}_{{\rm{sd}}}}\mathrm{(1}+A(E,Z,{\rm{\Delta }})-B(E,Z,{\rm{\Delta }}))dE,$$

where *f*(*E*),*T*,Δ and *Z* are the Fermi-Dirac distribution function, temperature, superconducting gap and the dimensionless parameter representing the potential barrier in the junction, respectively. *A*(*B*) is the probability of the equal-spin AR (normal reflection) defined in the BTK theory^34^. However, this standard BTK theory cannot explain the coexistence of side peaks and sub-gap feature as observed in Fig. 2(b) and (c). Herein, to apply the BTK theory for such cases, we assume two different transport channels in the proximity region, labeled channel *α* in which the equal-spin AR occurs and channel *β* with no spin-flip process. A schematic representation of the transport in these channels is shown in Fig. 3(a). The channel *α* can generate the sub-gap features reflecting the conductance enhancement due to the equal-spin AR, while the channel *β* can generate the side peaks reflecting the quasiparticle peaks of the superconducting bulk gap energy via normal reflection. Then, the normalized differential conductance of the junction can be written as

2 $$P\times {G}_{{\rm{int}}}^{\alpha }({V}_{{\rm{sd}}},{Z}_{\alpha },T,{{\rm{\Delta }}}_{\alpha })+\mathrm{(1}-P)\times {G}_{{\rm{int}}}^{\beta }({V}_{{\rm{sd}}},{Z}_{\beta },T,{{\rm{\Delta }}}_{\beta }),$$

where ${G}_{{\rm{int}}}^{\alpha },{G}_{{\rm{int}}}^{\beta },{Z}_{\alpha },\,{\rm{and}}\,{Z}_{\beta }$ are the normalized differential conductance of the channel *α*, the normalized differential conductance of the channel *β*, the parameter *Z* in the eqn. (1) of the channel *α* and that of the channel *β*, respectively. Δ_*α*_, and Δ_*β*_ are the proximity gap energy, and the bulk gap energy, respectively. Parameter *P* indicates the relative contribution of the two AR channels. Therefore, strong SOI can invoke more spin-flips to make larger the value of *P*. We note that the appearance of two superconducting gaps are theoretically discussed in the case of coexistence of spin-singlet and triplet superconducting pair amplitude^38,39^. We executed numerical calculations to fit the experimental data (see SI).

The best fitting result is shown in Fig. 3(b) by the solid lines plotted alongside the experimental data at 4.0 T. The obtained Δ_*α*_ and Δ_*β*_ are plotted as a function of *dI*/*dV* in Fig. 3(c). *dI*/*dV* for the x-axis is the differential conductance of the normal state measured at *V*_sd_ = 3.5 mV in units of *e*^2^/*h* and indexed by *g*. Δ_*β*_ is derived from the side peak positions and has a convex upward trend in each plateau-transition regime between plateaus of g = 0 and 1, 1 and 2, and 2 and 3, respectively. These Δ_*β*_ values are larger than the true bulk gap due to the dissipation in the QH bulk state (the equivalent circuit is the same as shown in Fig. 1(f)). We assume that the superconducting bulk gap Δ_*bulk*_ = 0.35 meV which is derived in Fig. 1(c) is unchanged with *V*_tg_ and therefore Δ_*β*_ in Fig. 3(c) should be equal to Δ_*bulk*_. This assumption is probably valid because the observed Δ_*β*_ in the plateau regime where there is no dissipation is consistent with Δ_*bulk*_ (see SI). Then we use the same equivalent circuit model as used for evaluating Δ_*bulk*_ to calibrate the value of Δ_*α*_ and finally obtain the true proximity gap of ${{\rm{\Delta }}}_{{\rm{triplet}}}\simeq 0.1\,{\rm{meV}}$ as 0.35 × Δ_*α*_/Δ_*β*_ shown in Fig. 3(c) (see SI for details).

We also derived the parameters *P* and *Z*_*α*_ and plot them as a function of change of *g* in Fig. 3(c), i.e. Δ*g* = 0 to 1 between plateaus in Fig. 3(d) and (e), respectively. The pink rectangles, blue triangles, and orange circles represent the parameters derived from the right, center, and left panel of Fig. 3(b), respectively. Figure 3(d) shows that the estimated *P* in each transition regimes has similar results. If the SOI of InAs QW is assigned to the spin-flip process, *P* can have the different value in different transition regime at the same magnetic field because top gate voltage can change not only the carrier density but also the Rashba SOI and the Zeeman energy (10 meV at 4 T) is large enough comparing to the SOI strength of InAs. Therefore, we think the strong SOI for the spin-flip process comes from the SOI of NbTi or the interfacial SOI between InAs and NbTi^40,41^. Indeed the spin-flip process in NbN is mentioned in the experimental report on junctions of NbN and graphene in strong magnetic field^42^. *P* indicating the proportion of AR in channel *α* has the maximum ($\simeq 1$) and *Z*_*α*_ has a minimum at ${\rm{\Delta }}g\simeq 0.7$. Our experimental results are obtained by the two-terminal conductance measurement. Therefore the bulk contribution to the channel *α* becomes maximum at a position displaced from Δ*g* = 0.5 and likely located between Δ*g* = 0.5 and 1. Indeed we find that the bulk contribution is maximal at ${\rm{\Delta }}g\simeq 0.7$ which is consistent with measurement results on a Hall-bar device (see SI). These results strongly and quantitatively support that channel *α* is comprised of the spin-resolved QH bulk state and the conductance enhancement originates from the equal-spin AR between the QH bulk state and the superconductor, while the channel *β* is assigned to conventional AR. These results imply that the equal-spin AR can occur even with strong SOI instead of inhomogeneous magnetization in the SC junctions while previous studies for spin-triplet superconducting proximity effect utilized the inhomogeneity. We note that the sub-gap feature and the side peaks have been theoretically predicted for the case of spin-singlet and spin-triplet superconducting proximity effect between a topological insulator and a spin-singlet superconductor with magnetic field^39^. There are a few theoretical works describing AR in the QH edge state^10,43–45^, but none focus on the QH state in the plateau-transition regime, and so further theoretical effort is necessary to reproduce the junction properties between superconductor and spin-resolved QH bulk state. From the topological aspects, theory predicts that the chiral topological superconductor state can be realized in superconductor-QH state junctions and therefore such junctions can be utilized to study non-Abelian statistics of the Majorana Fermions localized at the center of vortexes near the plateau-transition regime^21^. Our results indicate that it is possible to induce the proximity gap even in the spin-resolved QH state via equal-spin AR and so realize such chiral topological superconductivity.

In summary, we studied the transport properties of junctions between a NbTi superconductor and an InAs QW in the spin-resolved QH regime. We observed sub-gap features indicating Andreev transport arising from two channels. One equal spin Andreev reflection channel which produces peaks at zero bias, and a conventional Andreev reflection channel producing side peaks. These results indicate that junctions of NbTi and the InAs QW are a promising candidate to experimentally study the spin-triplet superconducting proximity effect in semiconductors and also topological superconductivity.

## Electronic supplementary material

Supplementary Information
