# Supplementary material for: Equal-Spin Andreev Reflection on Junctions of Spin-Resolved Quantum Hall Bulk State and Spin-Singlet Superconductor
Source: Sci Rep. 2018 Feb 22;8:3454. doi: 10.1038/s41598-018-21707-0 (PMC5823919; doi:10.1038/s41598-018-21707-0)
Supplement: Supplementary file 1 — Supplementary Information [file 41598_2018_21707_MOESM1_ESM.pdf]

Supplementary Information: Equal-Spin Andreev Reflection on Junctions of Spin-Resolved Quantum Hall Bulk State and Spin-Singlet Superconductor

Sadashige Matsuo,[1,*] Kento Ueda,[1] Shoji Baba,[1] Hiroshi Kamata,[1,2] Mizuki Tateno,[1] Javad Shabani,[3,4] Christopher J. Palmstrøm,[3,5,6] and Seigo Tarucha[1,2,†]

[1]*Department of Applied Physics, University of Tokyo, 7-3-1 Hongo, Bunkyo-ku, Tokyo 113-8656, Japan*

[2]*Center for Emergent Matter Science, RIKEN, Hirosawa 2-1, Wako-shi, Saitama 351-0198, Japan*

[3]*California NanoSystems Institute, University of California, Santa Barbara, CA 93106, USA*

[4]*Center for Quantum Phenomena, Physics Department, New York University, New York, NY 10003, USA*

[5]*Electrical and Computer Engineering, University of California, Santa Barbara, CA 93106 USA*

[6]*Materials Department, University of California, Santa Barbara, CA 93106, USA*

* matsuo@ap.t.u-tokyo.ac.jp

† tarucha@ap.t.u-tokyo.ac.jp

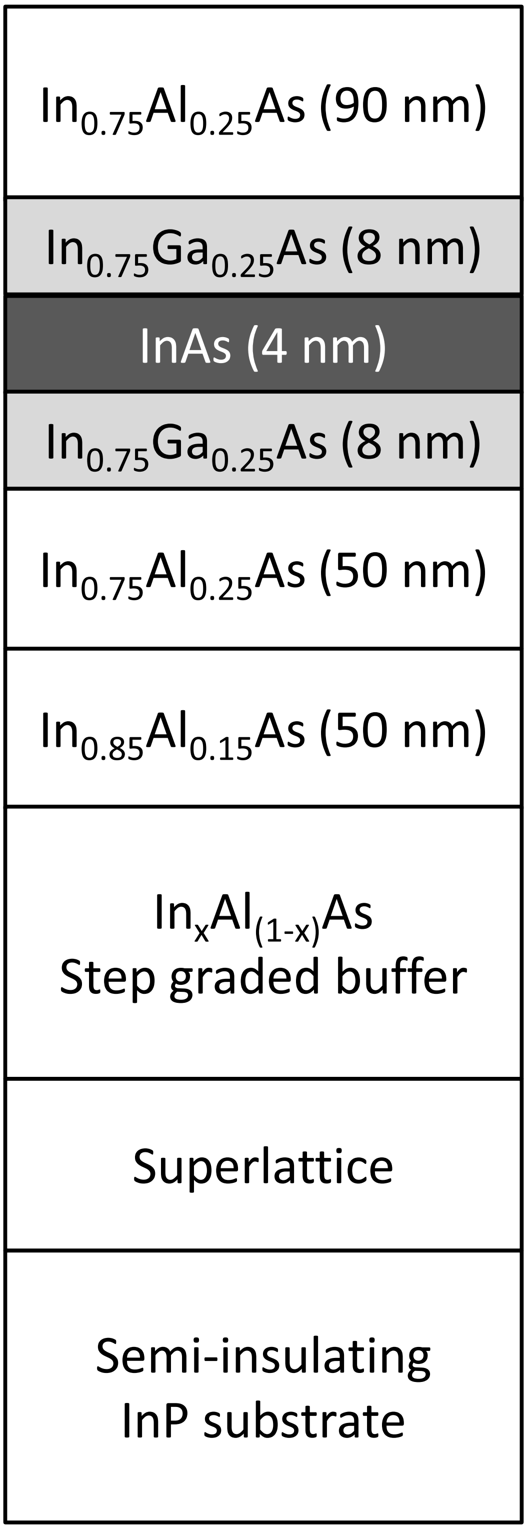

FIG. S1. Schematics of the InAs heterostructure material stack. The 2DEG exists in the 4 nm-thick InAs QW.

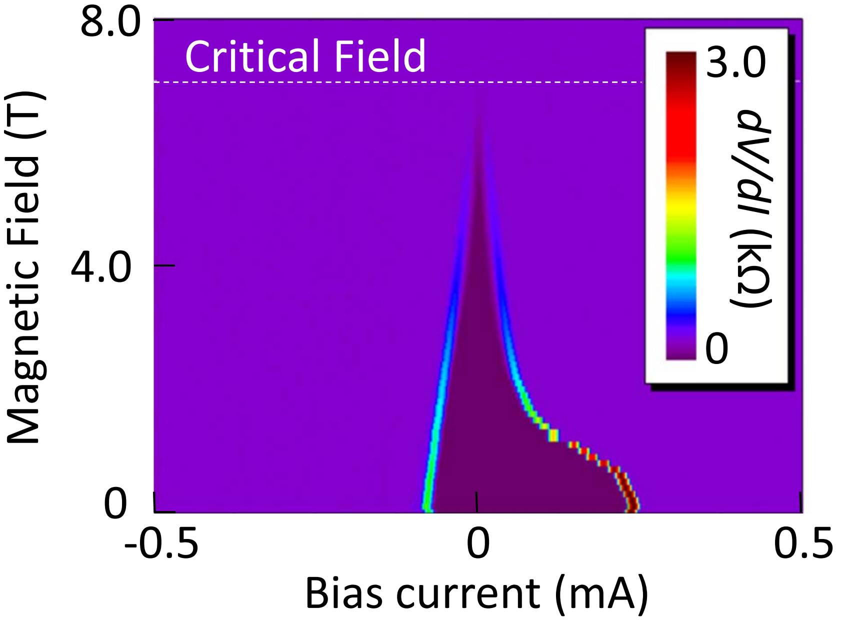

FIG. S2. Measurements of the critical field of a superconducting NbTi 150nm thin film. dV/dI as functions of magnetic field and bias current measured at 2.0 K. The NbTi holds superconductivity when dV/dI is equal to 0. The critical field is 7.0 T.

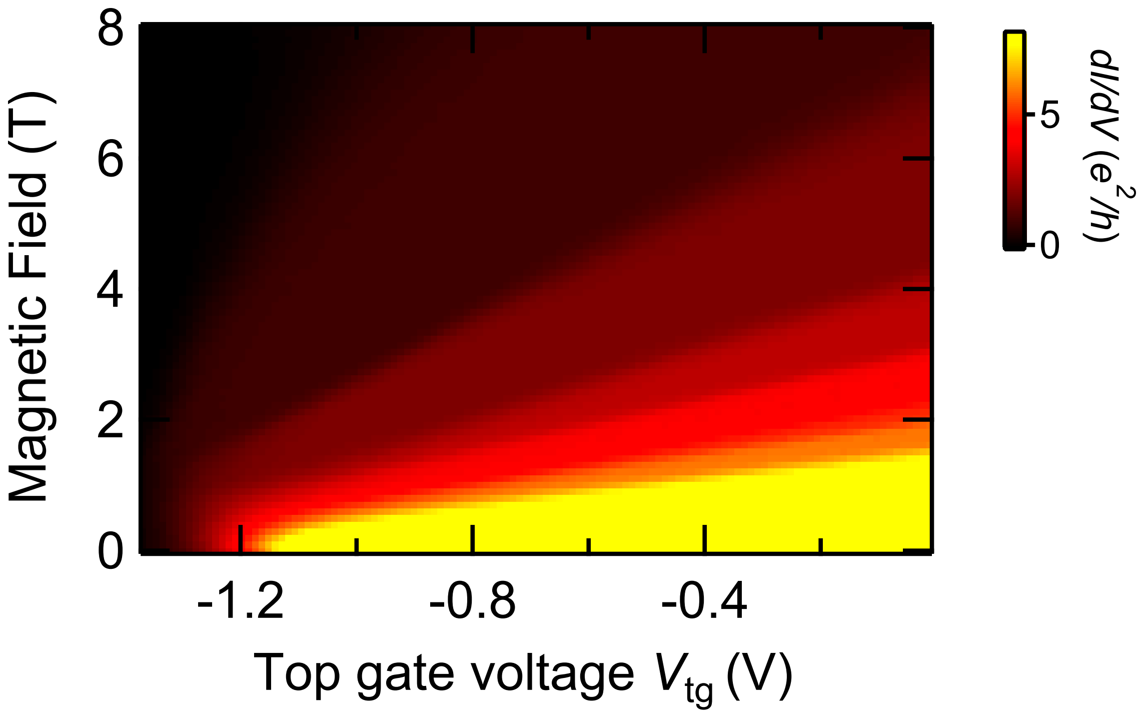

FIG. S3. Conductance as functions of magnetic field and top gate voltage.

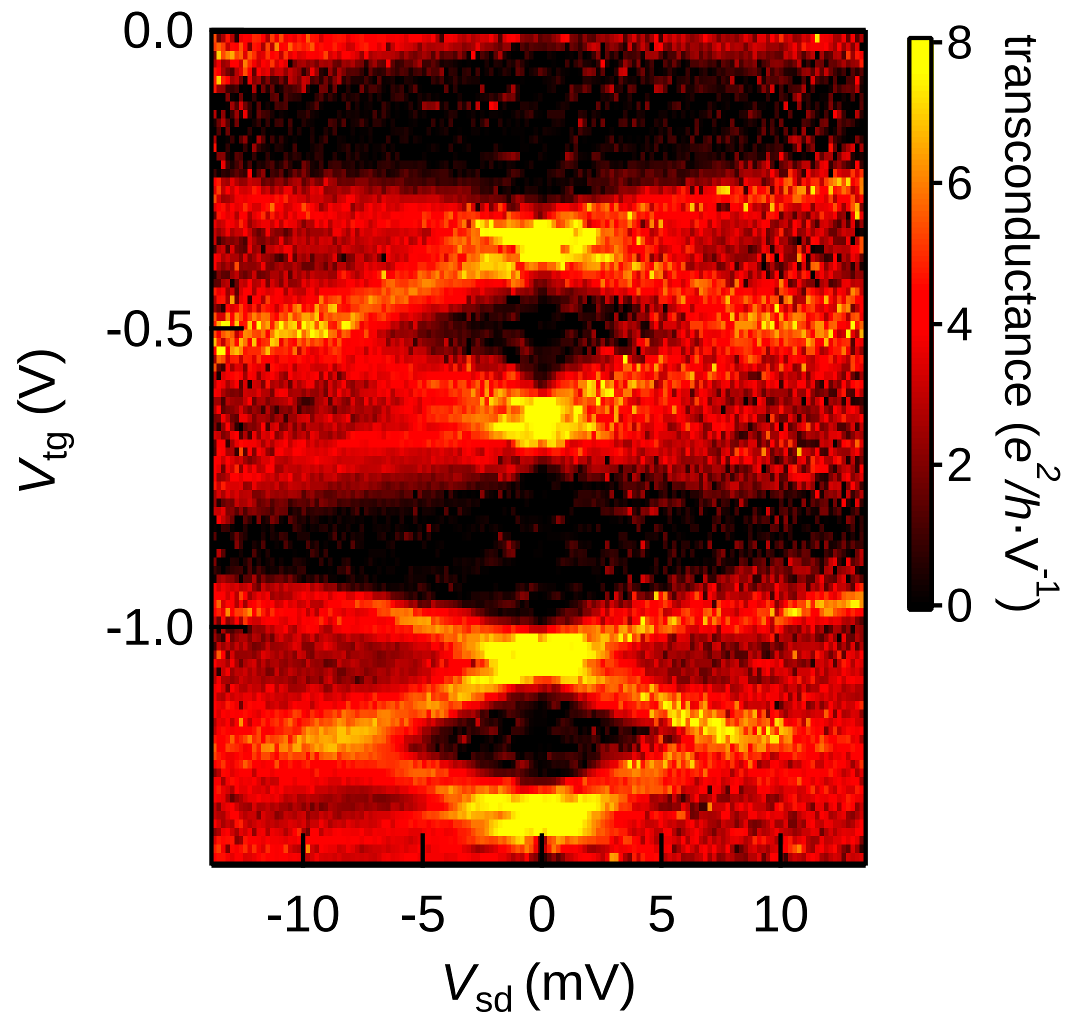

FIG. S4. Transconductance as functions of bias voltage and top gate voltage obtained at 2.4 T.

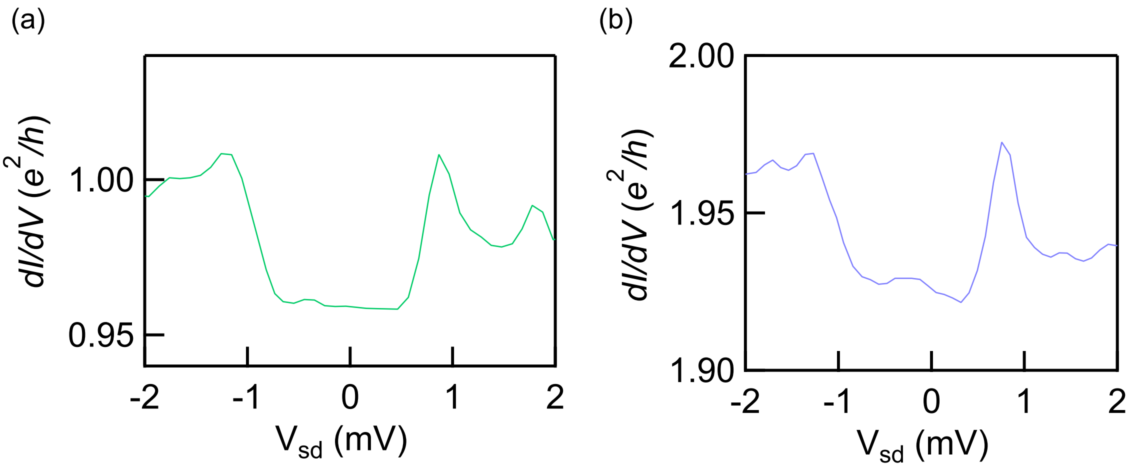

FIG. S5. Typical results of the dI/dV vs V_{sd} in the plateau regime obtained at 4 T are shown. The result on the $\nu = 1$ and 2 plateau is shown in (a) and (b), respectively.

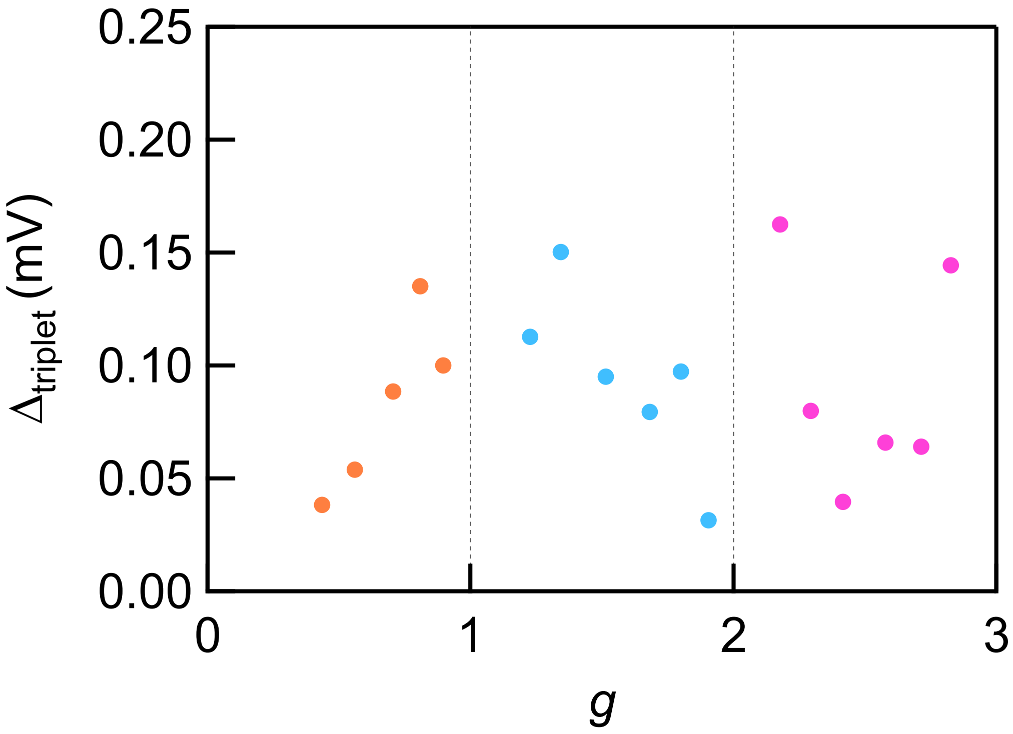

FIG. S6. The calculated spin-triplet superconducting proximity gap energy is shown. The values are ∼0.1 meV.

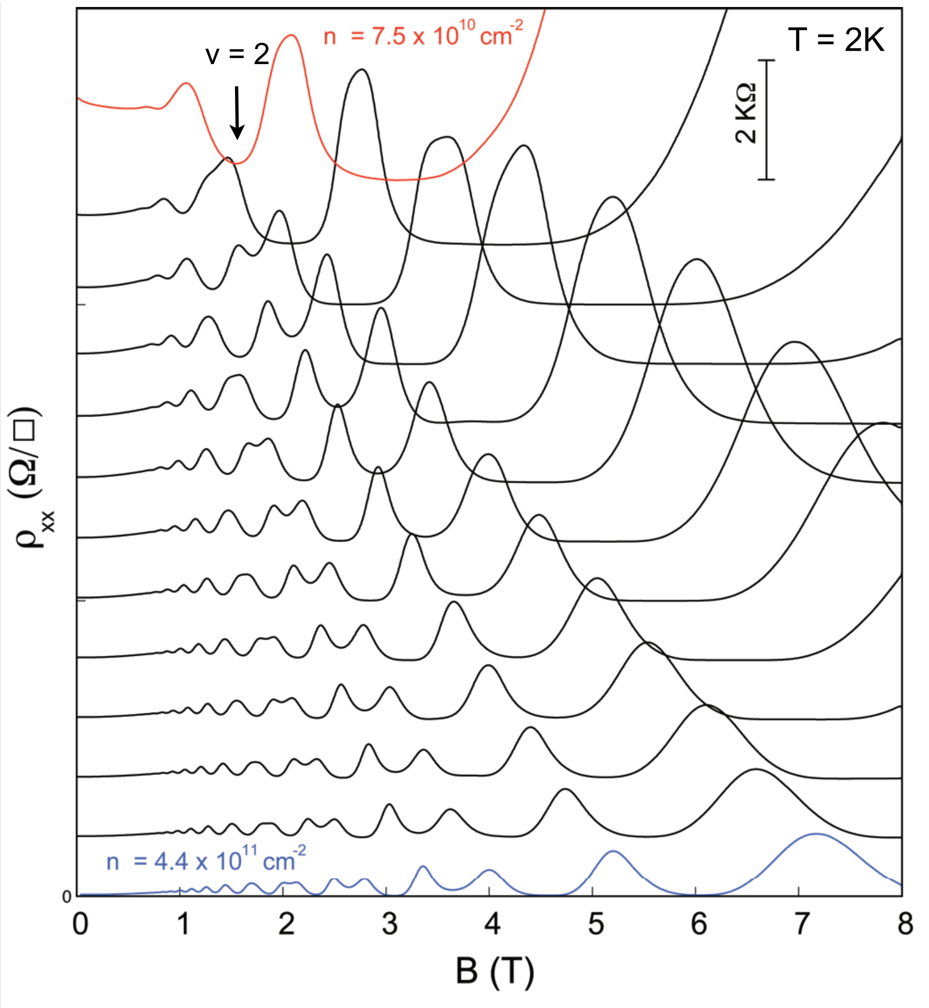

FIG. S7. The sheet resistance as a function of magnetic field is shown. The lines are incrementally shifted for clarity. We measured the resistance in the various carrier density by tuning the gate voltage. We subtract the peak height of the resistance around 4 T.

A. Device fabrication

We used an InAs QW grown by molecular beam epitaxy with the density 3×10^{11} cm^{-2} and the mobility 3×10^5 cm^{2}V^{-1}s^{-1}. The 2DEG is formed in the 4 nm-thick InAs layer. The material stack of the InAs heterostructure is schematically shown in Fig. S1. A mesa was first defined in the substrate by wet etching with an etchant based on H_3PO_4. Then, NbTi was sputtered to form the superconducting electrodes on the mesa edges, following a procedure of wet etching to make the clean edge exposed, sulfur passivation to avoid oxidization of the edge, and in-situ Ar plasma cleaning. Finally a gate electrode metal of Titanium and Gold was deposited on top to address the low QH filling regime even under a low magnetic field. The top gate is placed on an insulating layer made from cross-linked PMMA. This fabrication procedure creates no superconducting material on the top surface of the mesa. This is specially devised to control the carrier density not only of the mesa but also near the junction using the top gate voltage (discussed later). The two junctions are separated by 20 μm, so this device is assumed to have two independent contact regions.

B. NbTi superconductivity

To characterize the superconducting properties of the NbTi, we performed a current-bias measurement of the differential resistance dV/dI at various out-of-plane magnetic fields B for a 150 nm-thick NbTi thin film device at 2 K which is lower than the NbTi critical temperature of 6.5 K. The measured data shows a supercurrent branch as dV/dI=0 kΩ in dark purple near the zero current bias in Fig. S2. From this data, we evaluate the critical field of B =7.0 T. Herein, the coexistence of superconducting state and QH states can be realized if the 2DEG is in the spin-resolved QH regime for $B < 7.0$ T.

C. Superconducting proximity at 0 T

In Fig. 1(c) in the main text, there are dip structures around $V_{\rm sd} = \pm 0.7$ mV. These dip structures cannot be expected from the normal BTK model. These dip structures have also been reported in experimental studies of junctions of three dimensional topological insulators and superconductor [1]. Some theoretical works predict existence of spin-triplet superconducting proximity on such junctions which generate the dip structures [2, 3]. In our

case, strong spin-orbit interaction on the interface can affect the superconducting proximity even at 0 T and invokes finite spin-flip process. Therefore, we suspect that the dip structure may be related to the spin-triplet superconducting proximity even at 0 T.

About the superconducting bulk gap, the estimated Δ_{bulk} of 0.35 meV is smaller than the value of 0.99 meV, predicted from the critical temperature with conventional BCS theory. We think that this is due to degrade of NbTi in fabrication process of the top gate structure.

D. Transconductance in the QH regime

We measured the conductance as functions of magnetic field and top gate voltage in order to estimate quality of our InAs quantum Hall effect and Zeeman effect. Figure S3 shows the conductance as functions of B and $V_{\rm tg}$. As seen in this figure, quantized conductance plateaus appear at $B > 0.6$ T and the Zeeman splitting is found at $B >\sim 1$ T. 2.4 and 4 T is large enough to study the coexistence between the spin-resolved QH state and superconductor. Furthermore, we measured the transconductance defined by the deviation of dI/dV at 2.4 T. The results are plotted in Fig. S4. The diamond-shaped structure can be found. We focus on the bright areas, namely the plateau-transition regime in the main text.

Cyclotron energy, 20 meV can be estimated with effective mass, m=0.023m$_0$ at $B = 4.0$ T. Ratio of top gate voltage region of the 1st plateau and the sum of the 1st and 2nd plateaus is about 0.6 V/1.15 V. Therefore we can estimate the Zeeman energy of 10 meV at $B = 4.0$ T.

E. Sub-gap feature in QH plateau regime

In the main text, we focus on the sub-gap feature in QH plateau-transition regime. In this section, we show the measured dI/dV vs $V_{\rm sd}$ in the plateau regime. The typical results obtained at 4 T are shown in Fig. S5. Panel (a) and (b) indicates the result on the $\nu = 1$ and 2 plateau, respectively. Even in the plateau regime, there is a sub-gap feature with a small dip or gap-like structure. These results are similar to the sub-gap features observed for $\Delta g < 0.4$ of Figs. 2(b) and (c) in the main text. The estimated gap from these sub-gap features are nearly equal to the 0.35 mV calculated from the superconducting gap at 0 T. This supports our assumption in the main text that the spin-singlet superconducting

proximity gap at 4 T is the same as the superconducting gap obtained at 0 T. We conclude that the QH edge states don't contribute to the Andreev reflection in the device.

We now discuss why there is huge difference between the edge and the bulk in the AR signals. In the QH edge transport, the Andreev edge state is formed on the interface according to the theoretical prediction [4]. This Andreev edge state can classically be understood as sequential ARs with skipping orbit along the interface. (In our case, this sequential ARs should be the sequential equal-spin ARs.) Therefore, the reflected particle into the QH edge state depends on the number of ARs and can be an electron. However, AR occurs only once and reflects the hole in the QH bulk transport so it is expected the conductance enhancement due to single AR in the bulk transport.

F. Numerical calculation

We executed the numerical calculation using the model in which we assume the two channels, α and β. The fitting parameters are the superconducting gaps Δ_α and Δ_β, barrier strength Z_α ($Z_\beta = 1$), normal state interface conductance G_n, effective temperature ω, the relative contribution of channel α, P and offset conductance G_{offset}. We define $G_{\mathrm{int}}^{\alpha\beta}(V_{\mathrm{sd}}, Z_\alpha, T, \Delta_\alpha, \Delta_\beta)$ as eqn. (2) in the main text. Then the fitting function can be written as

$$\Big(\frac{1}{G_n \cdot G_{\mathrm{int}}^{\alpha\beta}(V_{\mathrm{sd}}, Z_\alpha, T, 2\Delta_\alpha, 2\Delta_\beta)} + \frac{1}{G_{\mathrm{offset}}}\Big)^{-1}.$$

We take care to confirm that our device has two independent superconducting-QH bulk state junctions. Therefore, the sub-gap features and the position in V_{sd} of the side peaks are consistent with $2\Delta_\alpha$ and $2\Delta_\beta$. To account for the effective temperature, we approximated the deviation of the Fermi-Dirac distribution function in eqn. (1) in the main text as the Gaussian function, $\exp(-((E - V_{\mathrm{sd}})/2\omega)^2)$, where ω is ideally equal to T but now ω includes the broadening due to inelastic scattering, inhomogeneity of the gap and the local heating [7, 8]. To execute the fitting, we constrict the fitting ranges for all the parameters, and especially we tightly constrict the Δ_α and Δ_β from the curve shapes. In order to reproduce the curve shape around the zero bias voltage, we changed the fitting range for each of the curves because the differential conductance of the 2DEG appears as background and the conductance has a large dependence on the bias voltage near the plateau regime. Due to this background dependence, we could not reproduce the curve shapes in the two lower curves of the left

panel and middle panel, and the lowest curve of the right panel in Fig.3(b) of the main text. In these cases, we evaluated only Δ_α and Δ_β from the sub-gap peak features ($2\Delta_\alpha$ and $2\Delta_\beta$ are indicated on the panels in Fig.3(b) as open and closed hexagons). Our fitting scheme includes many free parameters and results are sensitive to the constriction of the variable range. Additionally, the obtained errors for the parameters also depends on the constriction. However, the estimated gap energies and P produce relatively constant results with different fitting ranges, so we think it is valuable to discuss these parameters. All fits are executed with a genetic algorithm (GenCurvefit package for Igor Pro).

G. Calculation of the proximity gap energy

As written in the main text, we analyzed our experimental data with the model to evaluate Δ_α and Δ_β, the superconducting gap energies. However, these values are enlarged from the true bulk and proximity superconducting gap energies due to dissipation induced from the bulk state of the mesa. In the plateau regime, the transport is non-dissipative in the mesa, while the transport is dissipative in the plateau-transition regime due to the QH bulk state. Herein, in the plateau-transition regime, applied V_{sd} between two superconductors is divided into the voltage on the junctions and on the mesa, then the deduced Δ_β gives a larger gap energy as the contribution of the QH bulk state in the transport becomes larger. The equivalent circuit is represented in Fig. 1(f) in the main text. Consequently, Δ_β produces a peak in the middle of the plateau-transition regime where the bulk contribution becomes maximum. The true bulk superconducting gap energies (corresponding to the gap for channel β), 0.35 meV, can be evaluated from Δ_β near the plateau regime. From this gap energy, we calculated the true superconducting proximity gap energy, $\sim$ 0.1 meV as $0.35 \times \Delta_\alpha/\Delta_\beta$. The calculated gap, $\Delta_{\mathrm{triplet}}$ as a function of g, dI/dV at $V_{\mathrm{sd}} = 3.5$ mV in unit of e^2/h is shown in Fig.S6.

H. The maximum position of the bulk contribution

Our results for P and Z_α have a maximum and minimum, respectively, at $\Delta g \sim 0.7$. In this section, we estimate how large ρ_{xx} should be to make the bulk contribution have a maximum at $\Delta g \sim 0.7$.

As the shape of our device is square, the two-terminal conductance is written by $G_{2t} = \sqrt{\sigma_{xx} + \sigma_{xy}}$. σ_{xx} and σ_{xy} are the longitudinal conductivity and Hall conductivity [9–11], respectively. σ_{xx} has a maximum when the bulk contribution is maximum and the situation is given by $\sigma_{xy} = 0.5e^2/h + ne^2/h$ ($n = 0, 1, 2, 3...$), at which change in the filling factor $\Delta\nu$ is equal to 0.5. Herein, if the bulk contribution is maximum at $\Delta g \sim 0.7$ (namely $G_{2t} = 0.7e^2/h + ne^2/h$), $\sigma_{xx}(\sigma_{xy})$ should be $0.49e^2/h$ ($0.5e^2/h$), $0.80e^2/h$ ($1.5e^2/h$), and $1.02e^2/h$ ($2.5e^2/h$) in the $n = 0, 1,$ and 2 cases, respectively. From these conductivity, we can calculate the longitudinal resistance,$\rho_{xx} = \sigma_{xx}/(\sigma_{xx}^2 + \sigma_{xy}^2)$, resulting in $1.0h/e^2$ ($\sim$ 26kΩ), $0.28h/e^2$($\sim$ 7.0kΩ), and $0.14h/e^2$($\sim$ 3.6kΩ) in the $n = 0, 1,$and 2 cases, respectively. The calculated longitudinal resistance should be obtained when the bulk contribution is maximum, meaning ρ_{xx} is maximum with the calculated resistance in the region corresponding to the plateau-transition regime. We measured a Hall bar device at 2 K, fabricated from the same InAs quantum well wafer as we used for the superconducting devices and the results are shown in Fig. S7. ρ_{xx} has some peaks consistent with the finite bulk contribution. The maximum of ρ_{xx} at 4 T is at 4 kΩ and 1.5 kΩ corresponding to the $n = 1$and 2 cases, respectively. The measured resistances are comparable to the estimated resistance based on the assumption that the bulk contribution is maximum at $\Delta g \sim 0.7$ ($G_{2t} = 0.7e^2/h + ne^2/h$).

---

[1] M. Snelder, M. P. Stehno, A. A. Golubov, C. G. Molenaar, T. Scholten, D. Wu, Y. K. Huang, W. G. van der Wiel, M. S. Golden, and A. Brinkman, arXiv **1506**, 05923 (2015).

[2] P. Burset, F. Keidel, Y. Tanaka, N. Nagaosa, and B. Trauzettel, Phys. Rev. B **90**, 085438 (2014).

[3] P. Burset, B. Lu, G. Tkachov, Y. Tanaka, E. M. Hankiewicz, and B. Trauzettel, Phys. Rev. B **92**, 205424 (2015).

[4] H. Hoppe, U. Zülicke, and G. Schön, Phys. Rev. Lett. **84**, 1804 (2000).

[5] D. I. Pikulin, J. P. Dahlhaus, M. Wimmer, H. Schomerus, and C. W. J. Beenakker, New Journal of Physics **14**, 125011 (2012).

[6] A. D. K. Finck, D. J. Van Harlingen, P. K. Mohseni, K. Jung, and X. Li, Phys. Rev. Lett. **110**, 126406 (2013).

[7] G. T. Woods, R. J. Soulen, I. Mazin, B. Nadgorny, M. S. Osofsky, J. Sanders, H. Srikanth,

W. F. Egelhoff, and R. Datla, Phys. Rev. B **70**, 054416 (2004).

[8] J. P. DeGrave, A. L. Schmitt, R. S. Selinsky, J. M. Higgins, D. J. Keavney, and S. Jin, Nano Letters **11**, 4431 (2011), pMID: 21923114, http://dx.doi.org/10.1021/nl2026426.

[9] H. J. Lippmann and R. Kuhrt, Z. Naturforsch. A **13**, 462 (1958).

[10] H. H. Jensen and H. Smith, Journal of Physics C: Solid State Physics **5**, 2867 (1972).

[11] D. A. Abanin and L. S. Levitov, Phys. Rev. B **78**, 035416 (2008).
